# The Microstructure of GNR and the Mechanical Properties of Biobased PLA/GNR Thermoplastic Vulcanizates with Excellent Toughness

**DOI:** 10.3390/ma12020294

**Published:** 2019-01-18

**Authors:** Mingfeng Xia, Wenchao Lang, Yue Yang, Jihang Yu, Ningjing Wu, Qingguo Wang

**Affiliations:** 1 Key Laboratory of Rubber-Plastics, Ministry of Education/Shandong Provincial Key Laboratory of Rubber-Plastics, Qingdao University of Science & Technology, Qingdao 266042, China; 15764228967@163.com (M.X.); 17854269155@163.com (Y.Y.); a1260897171@163.com (J.Y.); qwang@qust.edu.cn (Q.W.); 2 School of Chemistry, Sun Yat-sen University, Guangzhou 510275, China; wenchao_lang@foxmail.com

**Keywords:** PLA, thermoplastic vulcanizates, bulk-grafting reaction, crosslinking density, super-tough, ductility

## Abstract

A series of different contents of glycidyl methacrylate (GMA)-grafted natural rubber (GNR) copolymers were fabricated via green bulk melt-grafting reactions, and super-tough bio-based poly (lactic acid) (PLA)/GNR thermoplastic vulcanizates (TPVs) were achieved by in-situ dynamic vulcanization. Increasing the graft yield, gel fraction, and crosslinking density of GNR vulcanizates effectively improved the ductility of the PLA/GNR TPVs, while prolonging the dynamic vulcanization time and increasing the GMA graft yield led to a notable enhancement in the impact toughness of the PLA/GNR TPVs. PLA/30 wt % GNR TPVs exhibited a significantly increased elongation (410%) and notched impact strength (73.2 kJ/m^2^), which were 40 and 15 times higher than those of the PLA/30 wt % NR TPVs, respectively. The new bio-based PLA/GNR TPVs offer promise as replacements for petroleum-based polymers in the automotive, 3D printing, and packaging fields.

## 1. Introduction

Poly (lactic acid) (PLA) is a promising bio-material with excellent biodegradability, good processing, and high mechanical strength, which makes it a suitable alternative for petrochemical polymers to alleviate the problems of diminishing oil resources. However, the disadvantages of PLA, including its brittleness and low heat distortion temperature, greatly limit its use [1,2,3,4]. Many flexible polymers, both biodegradable and non-biodegradable, have been blended with PLA to improve its toughness, including poly (butylene succinate), poly (butylene adipate-co-terephthalate), poly(carbonate), etc. [5,6,7]. Super-toughened PLA blends are currently the center of considerable attention [8,9,10,11,12,13,14]. Polypropylene/ethylene-propylene-diene rubber [15], poly (ethylene-n-butyl acrylate-glycidyl methacrylate) [16], polyurethane [17,18], unsaturated aliphatic polyester elastomer [19], polyester elastomer [20], poly (glycerol succinate-co-maleate) [21], natural rubber (NR) [22,23,24,25], and epoxidized natural rubber (ENR) [26] have all been used to improve the toughness of PLA via dynamic vulcanization. Bio-based PLA/35 wt % NR and PLA/40 wt % ENR TPVs both exhibit greatly improved impact strength, in which the vulcanizate phase presents a continuous network-like dispersion in the PLA matrix [22,26]. However, it is difficult to effectively enhance the performances of these PLA-based TPVs due to the difference in the polarity and rheological property between PLA and the rubber phase.

In our previous study, a kind of natural rubber modified by glycidyl methacrylate (NR-GMA) was prepared to decrease the polarity difference between PLA and NR. We focused on the effect of different contents of NR-GMA on the microstructure and toughness of PLA-based TPVs [27]. We found that the microstructure of NR graft-modified GMA (including graft yield, gel content, and crosslinking density) and vulcanization conditions had an important effect on the structure and performances of PLA-based thermoplastic vulcanizates (TPVs). We studied how to best control these factors to obtain the excellently toughened PLA TPVs. The influence of the microstructure of modified NR on the performance of PLA TPVs has not previously been systematically discussed. In this manuscript, three kinds of glycidyl methacrylate-grafted natural rubber (GNR) copolymers were synthesized, and the microstructure of these modified GNRs were characterized. The influence of the GNR microstructure and the vulcanizing conditions on the structure and properties of PLA TPVs were systematically investigated.

## 2. Materials and Methods

### 2.1. Materials

PLA (2003D, $\bar{M_{W}}$: 38 × 10^4^ g·mol^−1^, D: 1.59 g/cm^3^) was produced by Natureworks Co. (Minnetonka, MN, USA). NR (1#) was supplied by Jinlong Rubber Processing Co. (Shanggao, China). Glycidyl methacrylate (GMA) was supplied by Ruixiang Chemical Co. Ltd (Yangzhou, China). Dicumyl peroxide (DCP) was purchased from Yixiang Technology Co. Ltd. (Liuyang, China).

### 2.2. Preparation of the GNR

NR and different contents of GMA and DCP were mixed in a HAAKE torque rheometer with a 200 mL chamber size and roller rotor (Thermo Fisher Scientific, Waltham, MA, USA) at a temperature of 110 °C, and the rotor speed was set at 60 rpm. GNR copolymers with different graft yields and gel contents were prepared by the bulk graft reaction in accordance with the previous paper [27]. The formula and conditions are listed in Table 1.

The resultants were firstly extracted with acetone for 48 h to remove any unreacted GMA monomers, then the obtained GNR with acetone was dried in a vacuum oven for 24 h at 60 °C and weighted. The obtained GNR copolymer with acetone was refluxed at 135 °C in 50 mL of toluene for 4 h. It was added to 10 mL NaOH-CH_3_OH solution (0.05 mol/L) and back-titrated with trichloroacetic acid-methanol solution (0.05 mol/L) to the end point, with phenolphthalein used as the indicator. The GMA graft yield in GNR was calculated as follows:

(1) $$GMA\;graft\;yield\;\left(\%\right)=\frac{C\;\times \;(V_{0}-V)}{1000\;\times \;m_{1}}\times 142.15\times 100\%$$

where *m_1_* is the mass of GNR obtained with acetone, *C* is the concentration of trichloroacetic acid-methanol standard solution; and *V_0_* and *V* are the titration volume and back titration volume of the standard solution, respectively. M_GMA_ = 142.15 g/mol.

The extracted GNR with acetone was mixed with toluene at 135 °C for 24 h to remove the NR and NR-grafted-GMA copolymer. The insoluble gel product was dried in vacuum oven for 24 h at 80 °C and weighted, and referred to as *m_2_*. The gel fraction of GNR was calculated as follows:

(2) $$Gel\;fraction\;\left(\%\right)=\frac{m_{2}}{m_{1}}\times 100\%$$

where *m_2_* is the mass of residual GNR gel extracted with toluene.

### 2.3. Preparation of the PLA/GNR TPVs

The PLA and GNR (weight ratio: 70:30 wt/wt) were completely mixed in a HAAKE torque rheometer with a 200 mL chamber size and roller rotor at a rotor speed of 60 rpm from 135 to 160 °C for 10–15 min, after which DCP (1.5 wt % of GNR) was added, and the dynamic vulcanization was carried out for 5–10 min. The formula and preparation conditions are listed in Table 2. The PLA TPVs were hot-pressed at 180 °C and cut according to standard dimensions.

### 2.4. Characterization

Fourier transform infrared spectroscopy (FT-IR) spectra of the extracted GNR samples were recorded with a Bruker Tensor 27 Spectrometer (Bruker, Germany) using the attenuated total reflectance model with a resolution of 4 cm^−1^ for 32 scans.

^13^C nuclear magnetic resonance (NMR) spectra of the sample were obtained using a Varian Unity Inova Spectrometer (500 MHz) (Advance 2B, Brucker, Germany).

Mooney viscosity of NR and GNR were measured at 100 °C using a Mooney Viscosity Apparatus (EKT-2000M type, EKTRON TEK Corp. Ltd. Company, Qingdao, China).

The tensile properties of PLA-based TPVs were measured on a screw-driven universal tester (AL-7000M, Taiwan Gotech. Testing Machines Inc., Taichung, Taiwan) according to the ASTM D882 standard using a cross-head rate of 50 mm/min, and the notched Izod impact strengths of samples were measured on an impact tester (Suzhou Ligao Detection Equipment Co. Ltd., Suzhou, China) according to ISO 180.

The cross-linking densities (XLDs) of NR and GNR after dynamic vulcanization were measured using a nuclear magnetic resonance cross-link density spectrometer (NMR-CDS, 3500-DⅡ type, Innovative Imaging Corp. Company, Blieskastel, Germany). Dynamic mechanical analysis (DMA) of PLA TPVs was carried on a DMA instrument (Model Q800, TA Instruments, New Castle, DE, USA). from −100 to 150 °C at a heating rate of 3 °C /min, at a frequency of 1 Hz and at a 1% constant strain oscillation amplitude.

The morphologies of PLA based TPVs after tensile testing and impact testing were observed by scanning electron microscopy (SEM) (JSM-6700F, Japan Electronics Corp., Zhaodao, Tokyo, Japan). The cryo-fractured surfaces, impact-fractured surface, and tensile section were uniformly sputter-coated with a thin layer of platinum prior to examination. The average size of the vulcanizate particles in PLA/GNR TPVs was measured using Nano Measurer software 1.2.

## 3. Results

### 3.1. Preparation and Characterization of GNR and PLA/GNR TPVs

**Figure 1:**
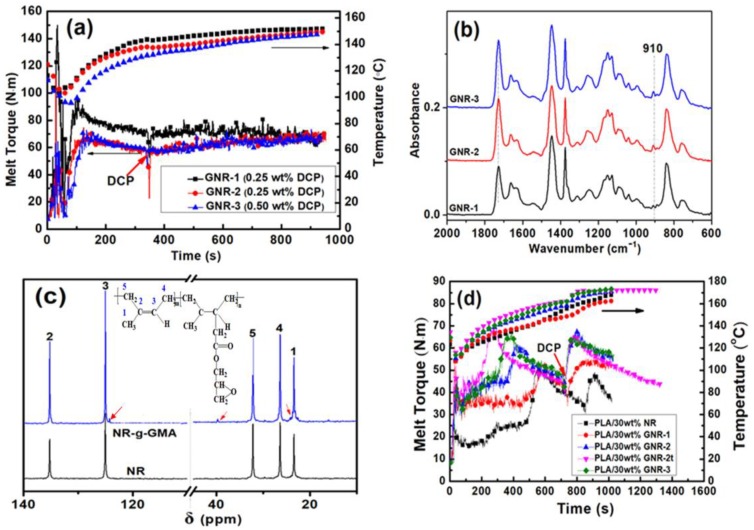
(**a**) Melt torque vs. time curves of GNR; (**b**) FT-IR spectra of GNR copolymers; (**c**) ^13^C-NMR spectra of NR-g- glycidyl methacrylate (GMA) and NR; (**d**) Melt torque vs. time curves of PLA/GNR thermoplastic vulcanizates (TPVs).

The evolution of NR bulk-grafted by GMA is shown in Figure 1a. After incorporating 5.0 wt % GMA, the melt torque of GNR-1 dramatically increased and reached a peak value at 100 s, after which the melt torque gradually decreased. After the addition of DCP, the melt torque gradually increased, indicating the occurrence of a graft reaction. The melt torque values of GNR-2 were lower than those of GNR-1 due to a higher GMA concentration of 14 wt %. With an increase of DCP (0.5 wt %), the melt torque of GNR-3 increased and gradually trended towards that of GNR-2, which may be attributed to the additional crosslinking reaction of the NR chains.

FT-IR spectra of GNR copolymers are shown in Figure 1b. The peaks of GNR at 1728, 1150, and 910 cm^−1^ corresponded to the stretching vibration of the ester group and the characteristic absorption of epoxy group of GMA, respectively. The ^13^C NMR spectra of the NR-g-GMA and NR are compared in Figure 1c, and the enlarged spectra are shown in Figure S1. NR (δ: 1CH_3_, 24.0 ppm; 2C = 135.2 ppm; 3CH = 125.0 ppm; 4CH-, 26.4 ppm; 5CH_2_-, 32.2 ppm). For the NR-g-GMA, new small peaks appeared at 39.77 (-C-) and 30.0 (-CH-) ppm, which were ascribed to the saturated double bonds of NR-g-GMA. The new small peaks at 124.3, 31.2, and 23.2 ppm corresponded to the isoprene segments adjacent to the grafted isoprene segments. The measurement results verified that GMA was grafted on the NR. The graft yield and gel content of the GNR copolymers were measured by titration and extraction.

The structural parameters of the GNR copolymers are shown in Table 3. The graft yield of GNR increased with the increase of GMA and DCP content. When the DCP concentration was increased to 0.5 wt %, the graft yield, gel fraction, and Mooney viscosity increased to 5.4%, 0.59, and 86.9 N·m, respectively. These results indicate that the graft reaction progressed further with an increase in the GMA monomer and DCP concentration, while more cross-linking reactions between NR and GMA resulted in the higher gel fraction and Mooney viscosity of GNR.

Figure 1d shows the preparation process of PLA/30 wt % GNR vulcanizates. The melt torque of PLA/30 wt % GNR-1 gradually increased and reached its first peak at 600 s. After the addition of 1.5 wt % DCP, the melt torque reached its second peak in the vulcanization stage. For the PLA/30 wt % GNR-2 and PLA/30 wt % GNR-3 vulcanizates, the melt torque in the vulcanization stage increased more rapidly, which was attributed to the higher graft yield and gel content of GNR-2 and GNR-3. However, when the dynamic vulcanization time was prolonged, the melt toque for PLA/30 wt % GNR-2t continuously decreased. This was attributed to a slight fracture of the crosslinking networks of the GNR vulcanizates under high shear.

### 3.2. Mechanical Properties of PLA/GNR Vulcanizates

**Figure 2:**
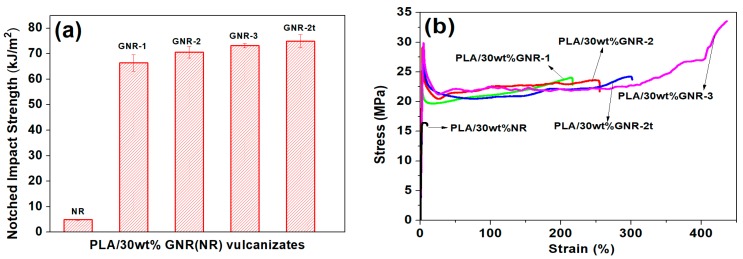
(**a**) Notched impact strengths and (**b**) The stress-strain curves of PLA/30 wt % GNR (NR) TPVs.

The mechanical properties of the PLA/30 wt % NRs and PLA/30 wt % GNRs are compared in Table 4. Figure 2a shows the notched impact strengths of PLA/30 wt % GNR(NR) TPVs. The notched impact strengths of PLA/30 wt % GNR-1 and PLA/30 wt % GNR-2 TPVs dramatically increased from 4.9 kJ/m^2^ of PLA/30 wt % NR TPVs to 66.4 and 70.6 kJ/m^2^, respectively. With the increase of GMA graft yield and gel fraction of GNR, the notched strength of PLA/30 wt % GNR-3 TPVs further increased to 73.2 kJ/m^2^, almost 14 times greater than that of PLA/30wt % NR TPVs. By prolonging the dynamic vulcanization time, the notched impact strength of PLA/GNR-2t TPVs increased to 74.9 kJ/m^2^.

For all the PLA/30 wt % GNR vulcanizates, a very clear yielding could be observed in the stress–strain curves (Figure 2b). The elongation of PLA/GNR-1 and PLA/30 wt % GNR-2 TPVs were dramatically increased from 12% of PLA/30 wt % NR TPVs to 197% and 240%, respectively. The elongation of PLA/GNR-3 TPVs further increased to 410%—almost 40 times greater than that of the PLA/30 wt % NR TPVs. The tensile properties of PLA/30 wt % GNR TPVs were obviously improved by increasing the GMA graft yield and gel fraction in the GNR copolymer.

### 3.3. Morphologies of PLA/GNR TPVs

**Figure 3:**
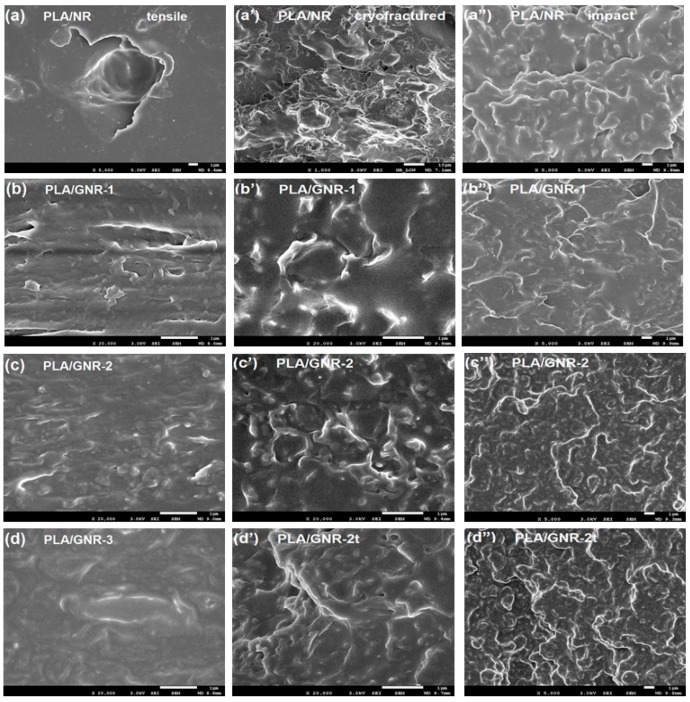
SEM images of PLA/30 wt % GNR(NR) TPVs at (**a**–**d**) tensile surface, (**a’**–**d’**) cryo-fractured surface, and (**a”**–**d”**) impact-fractured surface.

The tensile morphologies of the NR and GNR vulcanizates in PLA TPVs subject to tensile loading were compared by SEM images. For PLA/NR, large NR particles were debonded in the tensile testing direction, and voids gradually grew from the debonded NR particles, which was attributed to the weak interaction between the PLA matrix and NR particles (Figure 3a). Thus, the tensile strength and the elongation yield of the PLA/NR TPVs were both very low.

With an increase of GMA graft yield, the size of the GNR-1 and GNR-2 particles in PLA/30 wt % GNR-1 and PLA/30 wt % GNR-2 were obviously decreased, and most of the GNR-1 and GNR-2 particles were adhered at the PLA matrix, which indicated the improved interfacial interaction between the PLA matrix and GNR particles (Figure 3b,c). For the PLA/GNR-3 TPVs, nearly all of the GNR-3 particles were embedded in the PLA matrix (Figure 3d) and a wide range of shear yield in the PLA matrix was produced under tensile loading. Therefore, the excellent ductility of the PLA/GNR TPVs was attributed to the effects of the extensive shear yield of the PLA matrix and the tensile deformation of the GNR phase resulting from the good interfacial interaction between the PLA and GNR vulcanizates.

Figure 3a’–d’ show the phase morphologies of the cryo-fractured surfaces of the PLA/30 wt % GNR (NR) TPVs. For the PLA/30 wt % NR, it was observed that irregular NR particles were distributed in the PLA matrix shown in Figure 3a’, with the average size of the NR particles being approximately 5 μm. With increasing GMA graft yield, the GNR-1 and GNR-2 particles in the PLA matrix became more regular in shape and smaller (Figure 3b’,c’), compared to that of the PLA/30 wt % NR (Figure 3a’). The distribution of the GNR-2t particles (Figure 3d’) became more uniform, while their average size decreased to approximately 100 nm. It was also observed that the interface between the PLA matrix and the GNR vulcanizates became more difficult to discern, indicating that the interfacial interactions between the GNR vulcanizates and the matrix became stronger as the vulcanization time increased.

Figure 3a”–d’’ show the phase morphologies of the impact-fractured PLA/30 wt % GNR(NR) TPVs. Compared to that of PLA/30 wt % NR (Figure 3a”), more obvious plastic deformation of the PLA matrix occurred at the rough impact-fractured surface of the PLA/30 wt % GNR-1 and PLA/30 wt % GNR-2 TPVs (Figure 3b”,c”). For the PLA/30 wt % GNR-2t TPVs, greater plastic deformation occurred (Figure 3d”), and nearly no GNR particles were debonded from the matrix. These results demonstrate that the chemical interfacial adhesion became stronger with an increased GMA graft yield and vulcanization time, and a large proportion of micro–nanoscale GNR vulcanizates embedded in the PLA/GNR TPVs induced extensive plastic deformation and yielding of the PLA matrix, thus contributing to a greatly enhanced impact strength.

### 3.4. Dynamic Mechanical Analysis (DMA) of PLA/GNR TPVs

**Figure 4:**
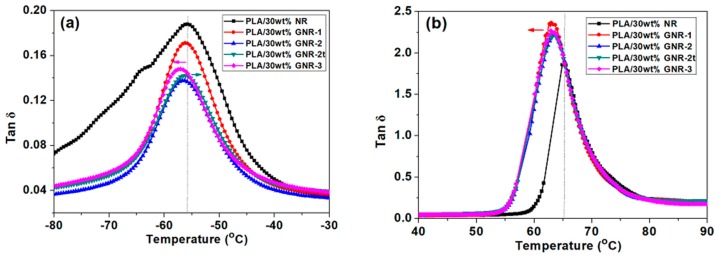
Dynamic mechanical analysis (DMA) curves of PLA/30 wt % NR and PLA/30 wt % GNR vulcanizates in the ranges of (**a**) −30–−80 °C, and (**b**) 40–90 °C.

Figure 4a,b show the DMA curves for the PLA/30 wt % GNR and PLA/30 wt % NR vulcanizates at the temperature ranges of −30–−80 °C and 40–90 °C, respectively. As shown in Table 5, for the PLA/30 wt % NR vulcanizates, the glass transition temperature (*Tg_1_*) of the NR vulcanizates and the *Tg_2_* of the PLA matrix were −55.8 °C and 61.5 °C, respectively. In the PLA/30 wt % GNR-1 vulcanizates, the *Tg_1_* of the GNR-1 vulcanizates decreased to −56.0 °C, while the *Tg_2_* of the PLA matrix decreased to 57.3 °C. With the increase of graft yield of GNR, the *Tg_2_* of the PLA/30 wt % GNR-2 vulcanizates decreased. This decrease in *Tg_2_* in the PLA matrix pointed to the occurrence of better interfacial compatibility between the PLA matrix and the GNR vulcanizates. The decreasing trend of *Tg_1_* in the GNR dispersed phase was attributed to the smaller dimensions of the vulcanizates in the PLA TPVs [14,27,28,29].

As the vulcanization time increased, the *Tg_1_* of the GNR-2t and the *Tg_2_* of the PLA matrix in the PLA/GNR-2t increased. These improvements in *Tg_1_* and *Tg_2_* were attributed to the increased interfacial reaction and crosslinking reaction with increasing dynamic vulcanization time.

### 3.5. Nuclear Magnetic Cross-Linking Measurement Analysis of GNR Vulcanizates

**Figure 5:**
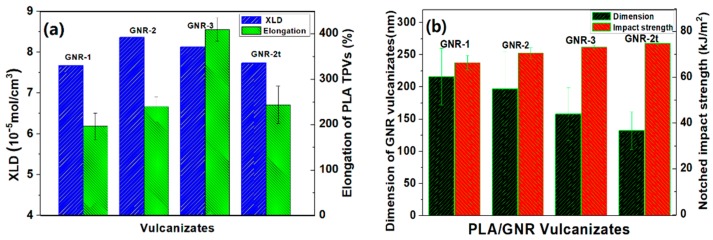
(**a**) Cross-linking densities (XLDs) of GNR vulcanizates and elongation at break of different of PLA/GNR TPVs, and (**b**) Dimension of GNR particles and notched impact strengths of different PLA/GNR TPVs.

To further investigate the mechanism leading to the super-toughness of the cross-linked GNR vulcanizates in the PLA TPVs, the cross-linking densities of the GNR vulcanizates were compared using nuclear magnetic cross-linking measurements [30]. Here, XLD represents the relative cross-linking densities of the GNR vulcanizates. As shown in Figure 5a, increasing the GMA graft yield of the GNR made the cross-linking density of the GNR-2 vulcanizates higher than that of the GNR-1 vulcanizates. However, the cross-linking density of the GNR-3 vulcanizates slightly decreased, which could be attributed to the slight degradation in the GNR-3 as a result of the free-radical graft reaction with a relatively high DCP content. Similarly, the cross-linking density of the GNR-2t decreased due to the partial breakage of the cross-linking network of the GNR-2 over a longer vulcanization time.

A relatively high cross-linking density (XLD: 8.13 × 10^−5^ mol/cm^3^) and a higher graft yield and gel content of the GNR-3 vulcanizates could produce an improvement in the elongation yield of the PLA/GNR-3 TPVs. However, increasing the vulcanization time did not obviously increase the elongation yield of the PLA/GNR-2t TPVs compared to that of the GNR-2 TPVs, due to the slight breakage of the cross-linking networks in the GNR-2t vulcanizates under longer vulcanization time.

As shown in Figure 5b, the size range of the GNR particles in the PLA TPVs was approximately 130–210 nm, and the dimensions of the GNR vulcanizates decreased as a result of increasing both the GMA graft yield of the GNR and the dynamic vulcanization time. The enhanced chemical interfacial interaction between the GNR vulcanizates and the PLA matrix promoted a further increase in the impact toughness of these PLA/GNR vulcanizates.

## 4. Conclusions

A series of GNR copolymers with different graft yields and gel contents were prepared via green bulk melt-graft reaction. A fine dispersion of micro–nanoscale GNR particles in the PLA matrix was achieved by improving the graft yields and gel fractions of the GNR and prolonging the vulcanization time. The elongation and notched impact strength of the PLA/30 wt % GNR-3 TPVs were significantly improved to 410% and 73.2 kJ/m^2^, respectively. GNRs with relatively higher cross-linking, graft yields, and gel contents exhibited a great improvement in the elongation of PLA/GNR TPVs. The higher graft yield and longer vulcanization time endowed the PLA/GNR TPVs with a greatly enhanced impact strength. These new bio-based PLA/GNR TPVs offer promise as potential replacements for petroleum-based polymers in the automotive, 3D printing, and packaging fields.

## Figures and Tables

**Table 1:** Formula of grafted natural rubber (GNR) copolymers via melt-grafting reaction.

Sample	GMA Content (wt %)	DCP (wt %)	Set temperature (°C)	Reaction Time (s)
GNR-1	5.0	0.25	110	600
GNR-2	14.0	0.25	110	600
GNR-3	14.0	0.50	110	600

**Table 2:** Formula of poly (lactic acid) (PLA)/GNR thermoplastic vulcanizates via dynamic vulcanization.

Sample	PLA (wt %)	NR (wt %)	GNR (wt %)	DCP of GNR (NR) (wt %)	Initial Set Temperature (°C)	Vulcanizing Time (s)
PLA/30 wt % NR	70	30	/	1.5	135	300
PLA/30 wt % GNR-1	70	/	30 (GNR-1)	1.5	135	300
PLA/30 wt % GNR-2	70	/	30 (GNR-2)	1.5	135	300
PLA/30 wt % GNR-3	70	/	30 (GNR-3)	1.5	135	300
PLA/30 wt % GNR-2t	70	/	30 (GNR-2)	1.5	135	600

**Table 3:** Characterization of GNR copolymer via melt-grafting reaction.

Sample	Graft Yield (%)	Gel Fraction	Mooney Viscosity (N·m)
GNR-1	2.3 ± 1.4	0.44 ± 0.05	60.1
GNR-2	5.0 ± 0.2	0.50 ± 0.01	68.8
GNR-3	5.4 ± 0.3	0.59 ± 0.02	86.7

**Table 4:** Mechanical properties of PLA/30 wt % GNR (NR) vulcanizates.

Sample	Notched Izod Impact Strength (kJ/m^2^)	Tensile Strength (MPa)	Elongation at Break (%)
PLA/30 wt % NR	4.9 ± 0.2	18.8 ± 0.5	12 ± 2
PLA/30 wt % GNR-1	66.4 ± 3.2	28.7 ± 0.4	197 ± 29
PLA/30 wt % GNR-2	70.6 ± 2.3	29.7 ± 0.5	240 ± 21
PLA/30 wt % GNR-3	73.2 ± 0.8	30.0 ± 1.0	410 ± 26
PLA/30 wt % GNR-2t	74.9 ± 2.6	27.5 ± 1.0	244 ± 41

**Table 5:** DMA data of PLA/NR and PLA/30 wt % GNR (NR) TPVs.

Sample	*Tg_1_* (°C)	*Tg_2_* (°C)
PLA/30 wt % NR	−55.8	61.5
PLA/30 wt % GNR-1	−56.0	57.3
PLA/30 wt % GNR-2	−56.6	57.0
PLA/30 wt % GNR-3	−57.1	56.9
PLA/30 wt % GNR-2t	−56.3	57.2

## Supplementary Materials

The following are available online at http://www.mdpi.com/1996-1944/12/2/294/s1, Figure S1: Enlarged 13C-NMR spectra of NR-g-GMA and NR.

## References

[1] Nagarajan V., Mohanty A.K., Misra M. (2016). Perspective on Polylactic Acid (PLA) based Sustainable Materials for Durable Applications: Focus on Toughness and Heat Resistance. ACS Sustain. Chem. Eng..

[2] Liu H.J., Zhang J. (2011). Research progress in toughening modification of poly (lactic acid). J. Polym. Sci. Part B Polym. Phys..

[3] Anderson K.S., Schreck K.M., Hillmyer M.A. (2008). Toughening polylactide. Polym. Rev..

[4] Yu L., Dean K., Li L. (2006). Polymer blends and composites from renewable resources. Prog. Polym. Sci..

[5] Ojijo V., Ray S.S., Sadiku R. (2013). Toughening of Biodegradable Polylactide/Poly (Butylene Succinate-co-adipate) Blends via in Situ Reactive Compatibilization. ACS Appl. Mater. Interfaces.

[6] Jiang L., Wolcott M.P., Zhang J. (2006). Study of Biodegradable Polylactide/Poly (Butylenes Adipate-co-terephthalate) Blends. Biomacromolecules.

[7] Yurye Y., Mohanty A.K., Misra M. (2016). Novel super-toughened bio-based blend from polycarbonate and poly (lactic acid) for durable applications. RSC Adv..

[8] Zeng J.B., Li K.A., Du A.K. (2015). Compatibilization strategies in poly (lactic acid)-based blends. RSC Adv..

[9] Dong W.Y., Jiang F.H., Zhao L.P., You J.C., Cao X.J., Li Y.J. (2012). PLLA microalloys versus PLLA nanoalloys: Preparation, morphologies, and properties. ACS Appl. Mater. Interfaces.

[10] Fang H.G., Jiang F., Wu Q.H., Ding Y.S., Wang Z.G. (2014). Supertough Polylactide Materials Prepared through In Situ Reactive Blending with PEG-based Diacrylate Monomer. ACS Appl. Mater. Interfaces.

[11] Lin L., Deng C., Lin G.P., Wang Y.Z. (2015). Super Toughened and High Heat-Resistant Poly (Lactic Acid) (PLA) Based Blends by Enhancing Interfacial Bonding and PLA Phase Crystallization. Ind. Eng. Chem. Res..

[12] Zhang K., Nagarajan V., Misra M., Mohanty A.K. (2014). Supertoughened renewable PLA reactive multiphase blends system: Phase morphology and performance. ACS Appl. Mater. Interfaces.

[13] Lin Y., Zhang K.Y., Dong Z.M., Dong L.S., Li Y.S. (2007). Study of hydrogen-bonded blend of polylactide with biodegradable hyperbranched poly (Ester Amide). Macromolecules.

[14] Liu H.Z., Song W.J., Chen F., Guo L., Zhang J.W. (2011). Interaction of microstructure and interfacial adhesion on impact performance of polylactide (PLA) ternary blends. Macromolecules.

[15] Chen Y.K., Xu C.H., Liang X.Q., Cao L.M. (2013). In situ reactive compatibilization of polypropylene/ethylene-propylene-diene monomer thermoplastic vulcanizate by zinc dimethacrylate via peroxide-induced dynamic vulcanization. J. Phys. Chem. B.

[16] He Y.S., Zeng J.B., Liu G.C., Wang Y.Z. (2014). Super-tough poly(l-lactide)/crosslinked polyurethane blends with tunable impact toughness. RSC Adv..

[17] Li Y., Shimizu H. (2007). Toughening of polylactide by melt blending with a biodegradable poly(ether) urethane elastomer. Macromol. Biosci..

[18] Kang H.L., Hu X.R., Li M.Q., Zhang L.Q., Wu Y.P., Ning N.Y., Tian M. (2015). Novel biobased thermoplastic elastomer consisting of synthetic polyester elastomer and polylactide by in situ dynamical crosslinking method. RSC Adv..

[19] Liu G.C., He Y.S., Zeng J.B., Li Q.T., Wang Y.Z. (2014). Fully biobased and supertough polylactide-based thermoplastic vulcanizates fabricated by peroxide-induced dynamic vulcanization and interfacial compatibilization. Biomacromolecules.

[20] Valerio O., Misra M., Mohanty A.K. (2017). Sustainable biobased blends of poly(lactic acid) (PLA) and poly(glycerol succinate-co-maleate) (PGSMA) with balanced performance prepared by dynamic vulcanization. RSC Adv..

[21] Chen Y.K., Yuan D.S., Xu C.H. (2014). Dynamically vulcanized biobased polylactide/natural rubber blend material with continuous cross-linked rubber phase. ACS Appl. Mater. Interfaces.

[22] Bitinis N., Verdejo R., Cassagnau P., Lopez-Manchado M.A. (2011). Structure and properties of polylactide/natural rubber blends. Mater. Chem. Phys..

[23] Bitinis N., Verdejo R., Maya E.M., Espuche E., Cassagnau P., Miguel A., Lopez-Manchado M.A. (2012). Physicochemical properties of organoclay filled polylactic acid/natural rubber blend bionano-composites. Compos. Sci. Technol..

[24] Mondal M., Gohs U., Wagenknecht U., Heinrich G. (2013). Polypropylene/natural rubber thermoplastic vulcanizates by eco-friendly and sustainable electron induced reactive processing. Radiat. Phys. Chem..

[25] Juntuek P., Ruksakulpiwat C., Chumsamrong P., Ruksakulpiwat Y. (2012). Effect of glycidyl methacrylate-grafted natural rubber on physical properties of polylactic acid and natural rubber blends. J. Appl. Polym. Sci..

[26] Wang Y.H., Chen K.L., Xu C.H., Chen Y.K. (2015). Supertoughened biobased poly (lactic acid)–epoxidized natural rubber thermoplastic vulcanizates: Fabrication, co-continuous phase structure, interfacial in situ compatibilization, and toughening Mechanism. J. Phys. Chem. B.

[27] Wu N.J., Zhang H., Fu G.L. (2017). Super-tough Poly(lactide) Thermoplastic Vulcanziates based on Modified Natural Rubber. ACS Sustain. Chem. Eng..

[28] Wu S.H. (1987). Formation of dispersed phase in incompatible polymer blends: Interfacial and rheological effects. Polym. Eng. Sci..

[29] Kfoury G., Raquez J.M., Hassouna F., Odent J., Toniazzo V., Ruch D., Dubois P. (2013). Recent advances in high performance poly(lactide): From “green” plasticization to super-tough materials via (reactive) compounding. Front. Chem..

[30] Litvinov V.M. (2006). EPDM/PP thermoplastic vulcanizates as studied by proton NMR relaxation: Phase composition, molecular mobility, network structure in the rubbery phase and network heterogeneity. Macromolecules.

