# Supplementary material for: The Microstructure of GNR and the Mechanical Properties of Biobased PLA/GNR Thermoplastic Vulcanizates with Excellent Toughness
Source: Materials (Basel). 2019 Jan 18;12(2):294. doi: 10.3390/ma12020294 (PMC6356512; doi:10.3390/ma12020294)
Supplement: Supplementary file 1 [file materials-12-00294-s001.pdf]

materials

MDPI

Article

The Microstructure of GNR and the Mechanical Properties of Biobased PLA/GNR Thermoplastic Vulcanizates with Excellent Toughness

Mingfeng Xia [1], Wenchao Lang [2], Yue Yang [1], Jihang Yu [1], Ningjing Wu [1,*] and Qingguo Wang [1]

[1] Key Laboratory of Rubber-Plastics, Ministry of Education/Shandong Provincial Key Laboratory of Rubber-Plastics, Qingdao University of Science & Technology, Qingdao 266042, China; 15764228967@163.com (M.X.); 17854269155@163.com (Y.Y.), a1260897171@163.com (J.Y.); qwang@qust.edu.cn (Q.W.)

[2] School of Chemistry, Sun Yat-sen University, Guangzhou 510275, China; wenchao_lang@foxmail.com (W.L.)

* Correspondence: ningjing_wu@qust.edu.cn

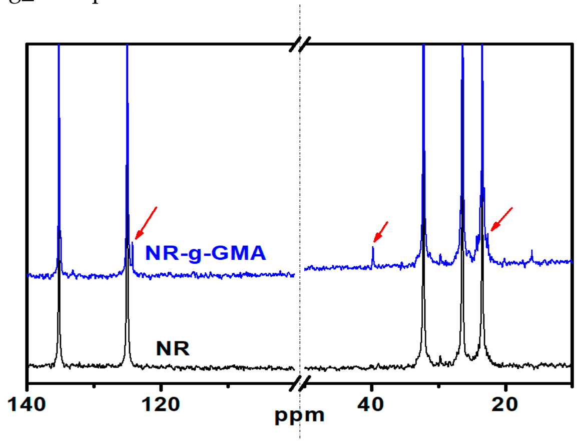

Figure S1. Enlarged ^{13}C-NMR spectra of NR-g-GMA and NR.

© 2019 by the authors. Submitted for possible open access publication under the terms and conditions of the Creative Commons Attribution (CC BY) license (http://creativecommons.org/licenses/by/4.0/).
